# Tyrosinase-based TLC Autography for anti-melanogenic drug screening

**DOI:** 10.1038/s41598-017-18720-0

**Published:** 2018-01-10

**Authors:** Kai-Di Hsu, Yu-Hin Chan, Hong-Jhang Chen, Shi-Ping Lin, Kuan-Chen Cheng

**Affiliations:** 1 Institute of Biotechnology, National Taiwan University, Taipei, 10617 Taiwan; 2 Graduate Institute of Food Science Technology, National Taiwan University, Taipei, 10617 Taiwan; 3 Department of Medical Research, China Medical University Hospital, China Medical University, Taichung, Taiwan

## Abstract

Tyrosinase-based TLC (thin layer chromatography) was developed for screening of anti-melanogenic drugs. In particular, this technique enables researchers to identify melanogenic inhibitor(s) in tested mixtures with the naked eye. In comparison with traditional colorimetric screening assays for tyrosinase inhibitor(s), not only is tyrosinase-based TLC a more cost-effective option (nearly one-tenth the enzyme cost of colorimetric methods) but also is a more sensitive detection approach for kojic acid (KA), a standard anti-melanogenic drug. The detection limit of tyrosinase-based TLC and colorimetric tyrosinase assay for KA was 0.0125 and 1.25 μg, respectively, demonstrating that the former was 100-fold more sensitive than the latter to determine the tyrosinase inhibitory rate of KA. Furthermore, the results of this method have demonstrated excellent precision by Gage Repeatability and Reproducibility (Gage R&R), with the variation of total Gage R&R being 28.24%. To verify the applicability of tyrosinase-based TLC, this platform was employed to screen melanogenic inhibitor(s) from *Ganoderma formosanum* extracts and two of all fractions (GFE-EA F4, F5) obtained showed depigmenting activity. It is noteworthy that these two fractions also exerted anti-melanogenesis activity on zebrafish, therefore verifying the credibility of tyrosinase-based TLC. In sum, this technique provides new insight into the discovery of novel melanogenic inhibitor(s).

## Introduction

Melanin is the major component responsible for skin pigmentation and protection of skin from UV-induced harms^1^. However, excessive melanin accumulation is considered aesthetically unfavorable, especially in Asian culture. Tyrosinase, a binuclear copper enzyme, is involved in melanin synthesis, which is responsible for catalyzing L-tyrosinase and 3, 4-dihydroxyphenylalanine (L-DOPA) to form melanin^2^. Consequently, the discovery of novel tyrosinase inhibitors for the development of anti-melanogenic drug has received great attention in the field of cosmetic medicine.

For anti-melanogenic drugs screening, various methods have been established from *in vitro* to *in vivo* assays. Currently, ultraviolet-induced hyperpigmentation in guinea pig and phenotype-based zebrafish are animal models both widely applied to evaluate the effects of candidate drugs for the inhibition of melanogenesis^3,4^. However, *in vivo* animal models often requires long experimental periods and extensive work, not to mention the considerable consumption of testing drugs used in animals for large-scale screening^5^. In view of the aforementioned disadvantages, *in vitro* assays such as cell-free mushroom tyrosinase activity assay, cell-based and MelanoDerm 3D tissue models^6,7^ are preferable to *in vivo* assays during preliminary screening of tyrosinase inhibitors from mixtures of natural products in terms of cost and efficiency^5^. Although these *in vitro* techniques provide effective approaches to evaluate depigmenting effects of unknown samples, the development of compound-oriented screening method is urgently required for the identification of bioactive compound(s). Taken together, positive results of both *in vitro* and *in vivo* anti-melanogenic assays merely demonstrate the existence of melanogenic inhibitor(s), but does not allow further isolation and purification of certain compound(s).

In our previous study, the ethyl acetate fraction of *Ganoderma formosanum* mycelium ethanolic extract (GFE-EA) had been demonstrated to inhibit melanin formation on the surface of zebrafish embryos^5^. We are currently working on the purification and identification of active compound(s) of GFE-EA. However, the considerable sample consumption used for anti-melanogenic evaluation by the aforementioned *in vitro* assays obstructed us to further purify and identify active compound(s) in GFE-EA. On the other hand, purification of the tested extracts without evaluating bioactivity beforehand results in excessive work. Therefore, we have developed a novel technique enabling the evaluation of both active compounds and bioactivity in GFE-EA.

Thin layer chromatography (TLC) is a simple method used widely in the separation of mixtures. With the association of various enzymes, TLC is currently considered an effective and practical technique used for the screening of novel bioactive compounds^8^. Several enzyme-based TLC platforms have been established to discover novel enzyme inhibitor(s), including lipase, acetylcholinesterase and beta-glucosidase inhibitor^9–11^. In this study, we have combined the evaluation of depigmenting activity with chromatographic separation, and developed a high-throughput enzyme-based TLC autographic system, which enables researchers to determine tyrosinase inhibitory activity and identify active compounds of a tested sample simultaneously.

## Results

### Efficacy comparison of L-tyrosine and L-DOPA for tyrosinase-based TLC bioautography

**Figure 1:**
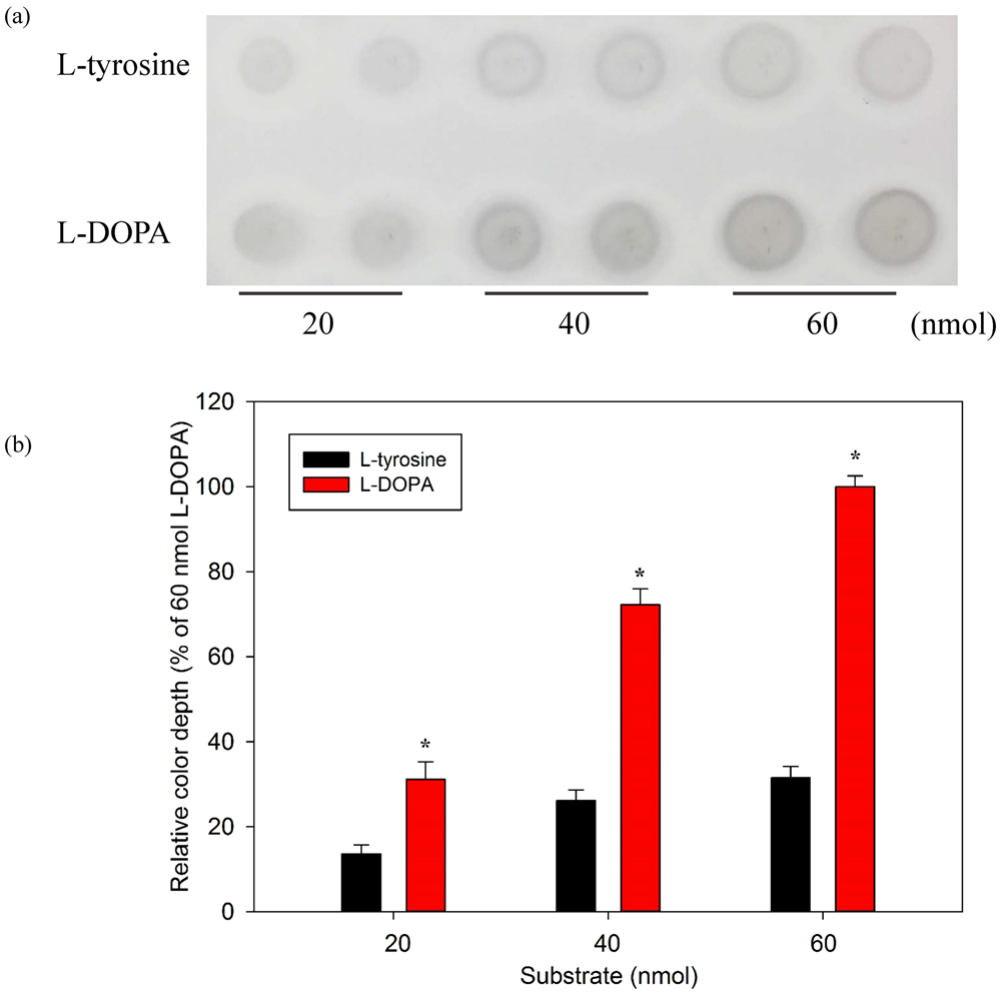
Efficacy comparison of L-tyrosine and L-DOPA for tyrosinase-based TLC bioautography after a 10-minutes reaction (amount of tyrosinase was 2 units per spot). (**a**) Results of various amounts of L-tyrosine and L-DOPA on tyrosinase-based TLC plate. (**b**) Quantification of melanin spots on TLC plates of 20, 40 and 60 nmol L-tyrosine and L-DOPA groups using ImageJ. *Indicates that the color depth of spots on TLC of L-DOPA groups are significantly higher than that of L-tyrosine groups (p < 0.05).

To establish a reliable tyrosinase-based TLC autography platform, effectiveness of L-tyrosine and L-DOPA were compared in this study. As shown in Fig. 1a, L-DOPA groups exhibited darker spot after a 10-minutes reaction with tyrosinase (2 units) than L-tyrosine groups with various amounts (20 to 60 nmol), which suggested that L-DOPA is preferable to L-tyrosine for observation by the naked eye. ImageJ, an image analysis software, was subsequently employed to quantify each dark spot on tyrosinase-based TLC. As compared to the L-DOPA groups, the dark depth of 20, 40 and 60 nmol of L-tyrosine groups were about 43.7, 36.2 and 31.5% of L-DOPA at the same amount, respectively (Fig. 1b). As a whole, L-DOPA was a more competent substrate than L-tyrosine for tyrosinase assay to present obvious melanin spot on TLC plate. Therefore, L-DOPA was employed to develop a tyrosinase-based TLC for anti-melanogenic drugs screening.

### Optimization of L-DOPA and tyrosinase amounts for tyrosinase-based TLC

**Figure 2:**
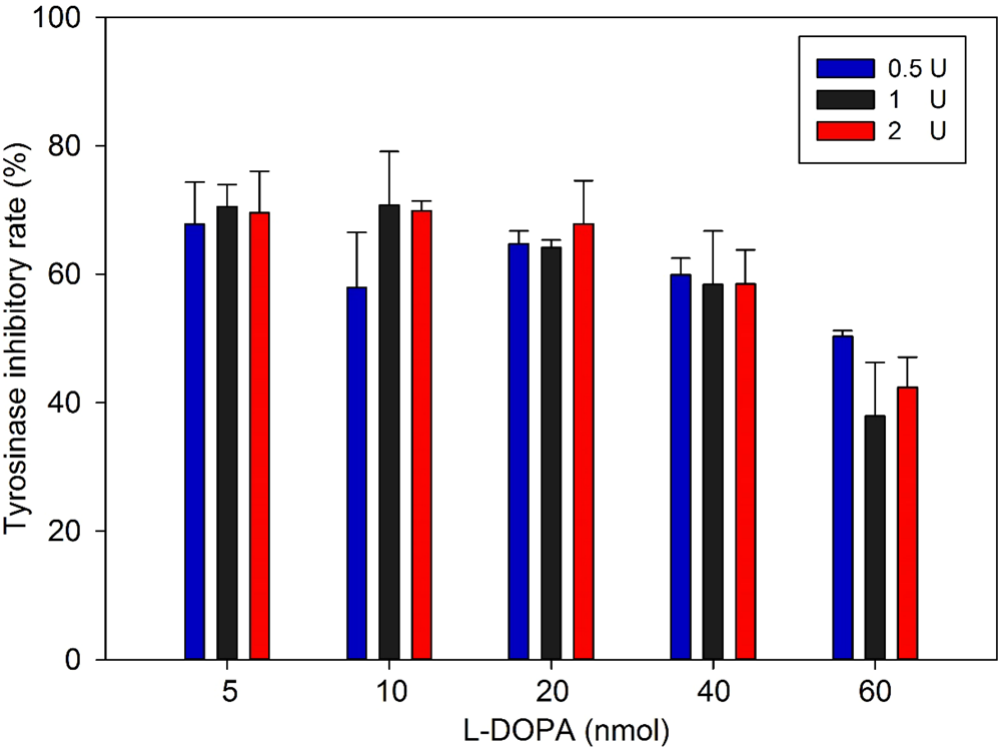
Optimization of L-DOPA and tyrosinase amount for tyrosinase-based TLC bioautography. Reactions were conducted with 0.5 μg kojic acid to evaluate suitable substrate and enzyme amount for this assay.

To establish a stable and sensitive assay platform for researchers, various amounts of L-DOPA (5–60 nmol) and tyrosinase (0.5–2 unit) were estimated to optimize tyrosinase-based TLC. Furthermore, kojic acid (0.5 μg) was used as a standard anti-melanogenesis drug to investigate sensitivity and stability of tyrosinase inhibitory assay under various amounts of L-DOPA and tyrosinase. As illustrated in Fig. 2, there was no significant difference between groups with different amount of tyrosinase. However, it was noteworthy that the high amount of L-DOPA groups (60 nmol) misled lower tyrosinase inhibitory activity of kojic acid compared to groups with 5 to 40 nmol L-DOPA. Furthermore, in view of the fact that groups with 20 nmol were more clearly observed by the naked eye than groups with 5 and 10 nmol L-DOPA (data not shown), and it was more economic than 40 nmol L-DOPA groups. Accordingly, 20 nmol L-DOPA was chosen for following tyrosinase-based TLC assay.

### A comparison of tyrosinase-based TLC with colorimetric tyrosinase assay on detection limit for kojic acid

**Figure 3:**
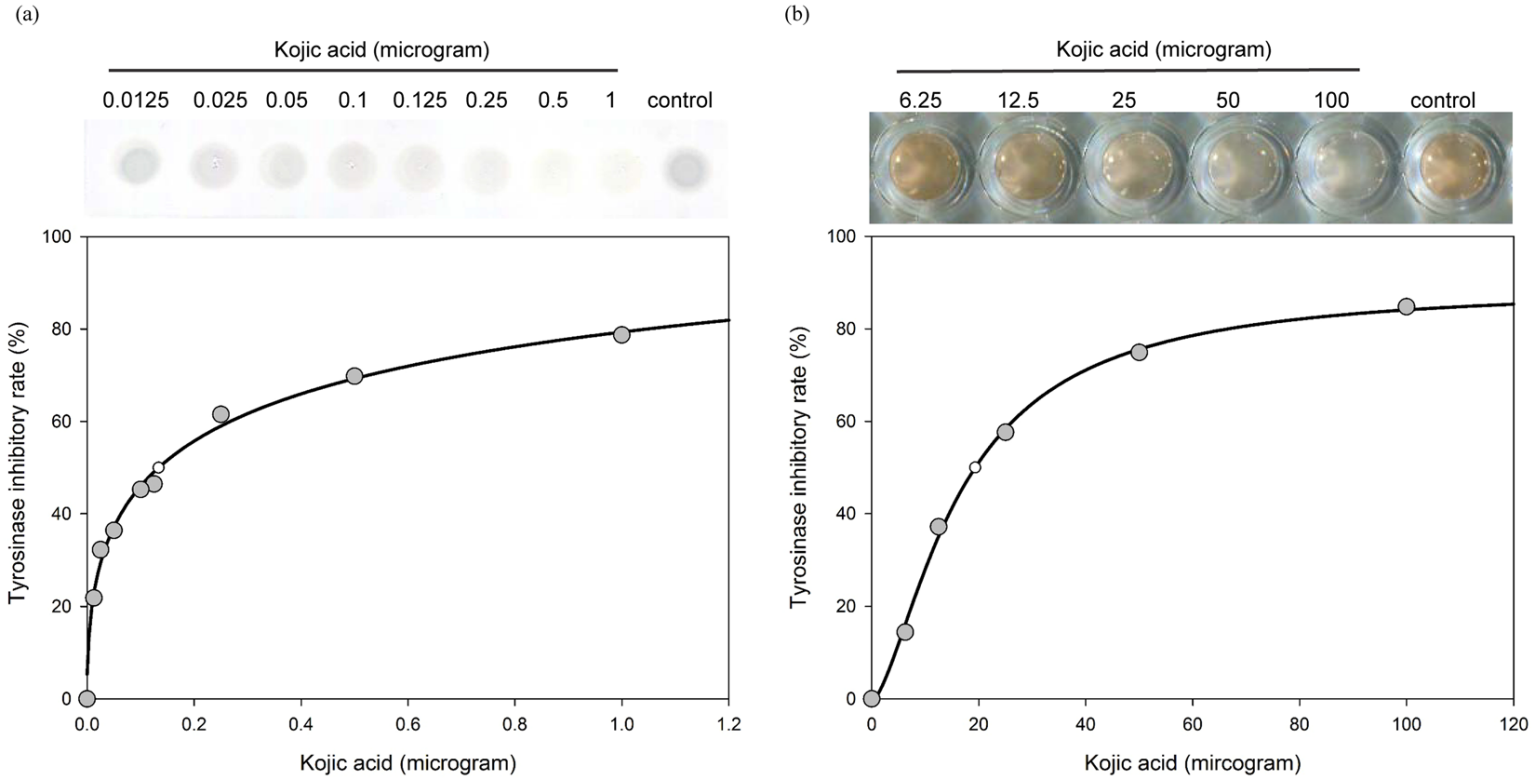
A comparison of tyrosinase-based TLC bioautography and colorimetric tyrosinase activity assay.

To reveal the sensitivity and detection limit of tyrosinase-based TLC, tyrosinase inhibitory activity of various amount of kojic acid was evaluated. Experimental results demonstrated the detection limit of tyrosinase-based TLC for KA was 0.0125 μg, which was 100-fold more sensitive than that of colorimetric tyrosinase assay (the detection limit for KA was 1.25 μg) (Fig. 3). These results demonstrated that tyrosinase-based TLC is more sensitive than traditional colorimetric method, suggesting that researcher can reduce consumption of tested compound(s) by tyrosinase-based TLC during bioactivity evaluation.

### Repeatability and reproducibility of tyrosinase inhibitory activity assay by tyrosinase-based TLC

**Figure 4:**
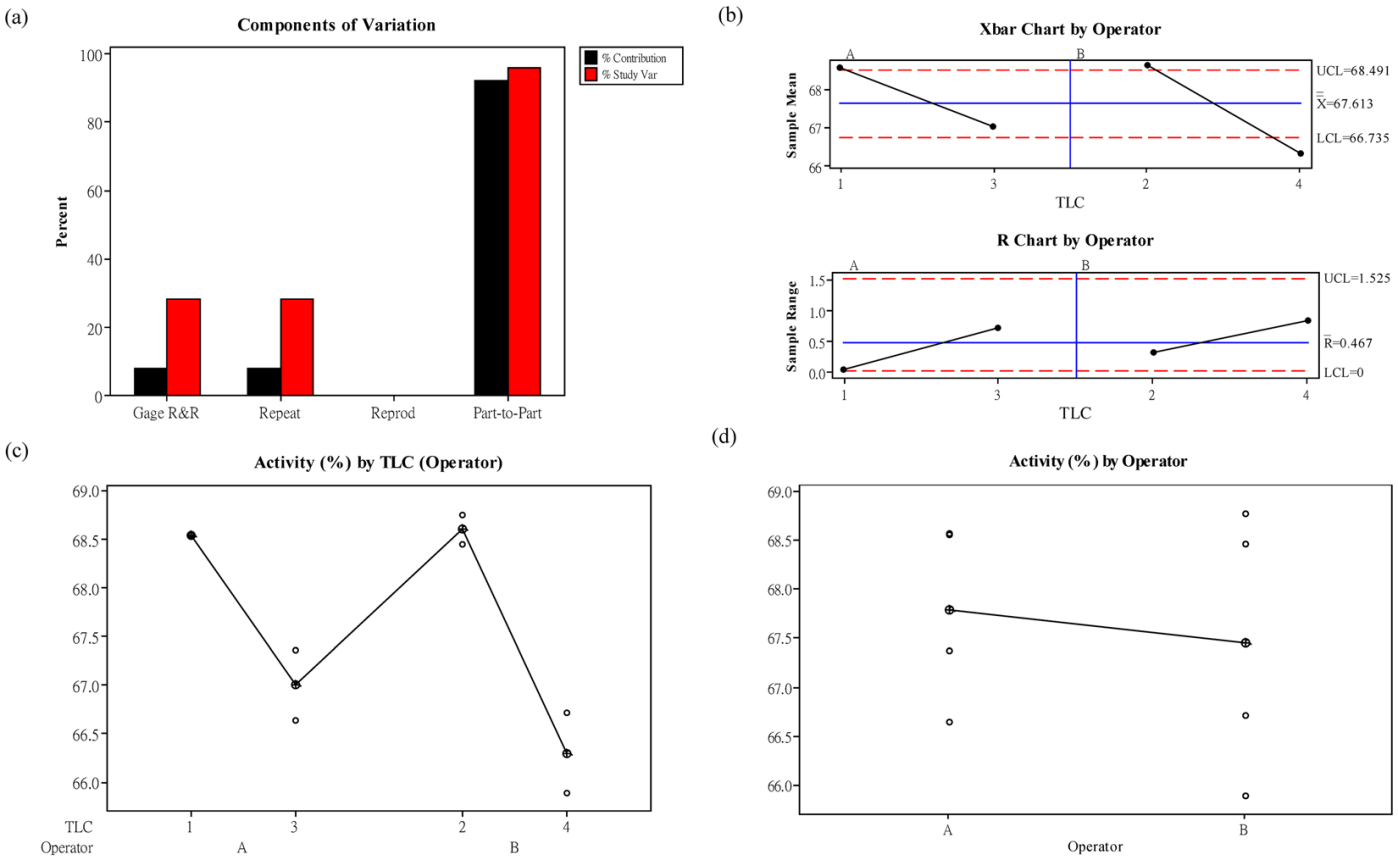
Gage R&R Nested study of Tyrosinase-based TLC. (**a**) Components of variation, (**b**) X-bar and R char by operator, (**c**) Measurements by TLC, and (**d**) Measurements by operator.

Tyrosinase inhibitory activity of KA (1 μg) was conducted by different operators to determine the repeatability and reproducibility of tyrosinase-based TLC by using Gage R&R Nested study. Result of operator p-value is 0.838, demonstrating that operator was not the source resulted in experimental variation (Table 1). Moreover, as illustrated in Fig. 4a, %study variation of part-to-part (instruments) and total Gage R&R (operator) was 95.93 and 28.24% (Table 2), respectively, indicating that operator is not a major factor caused variation. From X-char in Fig. 4b, it shows that values from several independent experiments vary more than the control limit (gird line), indicating that the difference of depigmenting activity of tested samples could be detected over endogenous error from experimental process. Furthermore, R-chart demonstrates that experimental results conducted by operator A and B was relatively consistent (Fig. 4b). As seen from the results of activity by TLC (operator) and activity by operator, the tyrosinase inhibitory activity of KA from two operators was similar at the range of 66–68% (Fig. 4c,d), verifying repeatability and reproducibility of this TLC bioautographic assay was stable.

**Table 1:** Analysis of variance of tyrosinase-based TLC assay conducted by different operators.

Source	DF^*^	SS^*^	MS^*^	F	P
Operator	1	0.20882	0.20882	0.0538	0.838
TLC (operator)	2	7.75596	3.87798	24.0768	0.006
Repeatability	4	0.64427	0.16107		
Total	7	8.60905			

*DF = Degrees of freedom; SS = Sun of squares; MS = Mean square.

**Table 2:** Gage R&R Nested analysis of tyrosinase-based TLC assay conducted by different operators.

Source	VarComp*	%Contribution (of VarComp)	SD* (6*SD)	SV*	%SV*
Total Gage R&R	0.16107	7.98	0.40133	2.40799	28.24
Repeatability	0.16107	7.98	0.40133	2.40799	28.24
Reproducibility	0.00000	0.00	0.00000	0.00000	0.00
Part-To-Part	1.85846	92.02	1.36325	8.17952	95.93
Total Variation	2.01952	100.00	1.42110	8.52660	100.00

Number of Distinct Categories = 4.

*VarComp = Variance component; SD = Standard deviation; SV = Study variation.

### Applicability of tyrosinase-based TLC to screen tyrosinase inhibitor from crude herbal mushroom extracts

**Figure 5:**
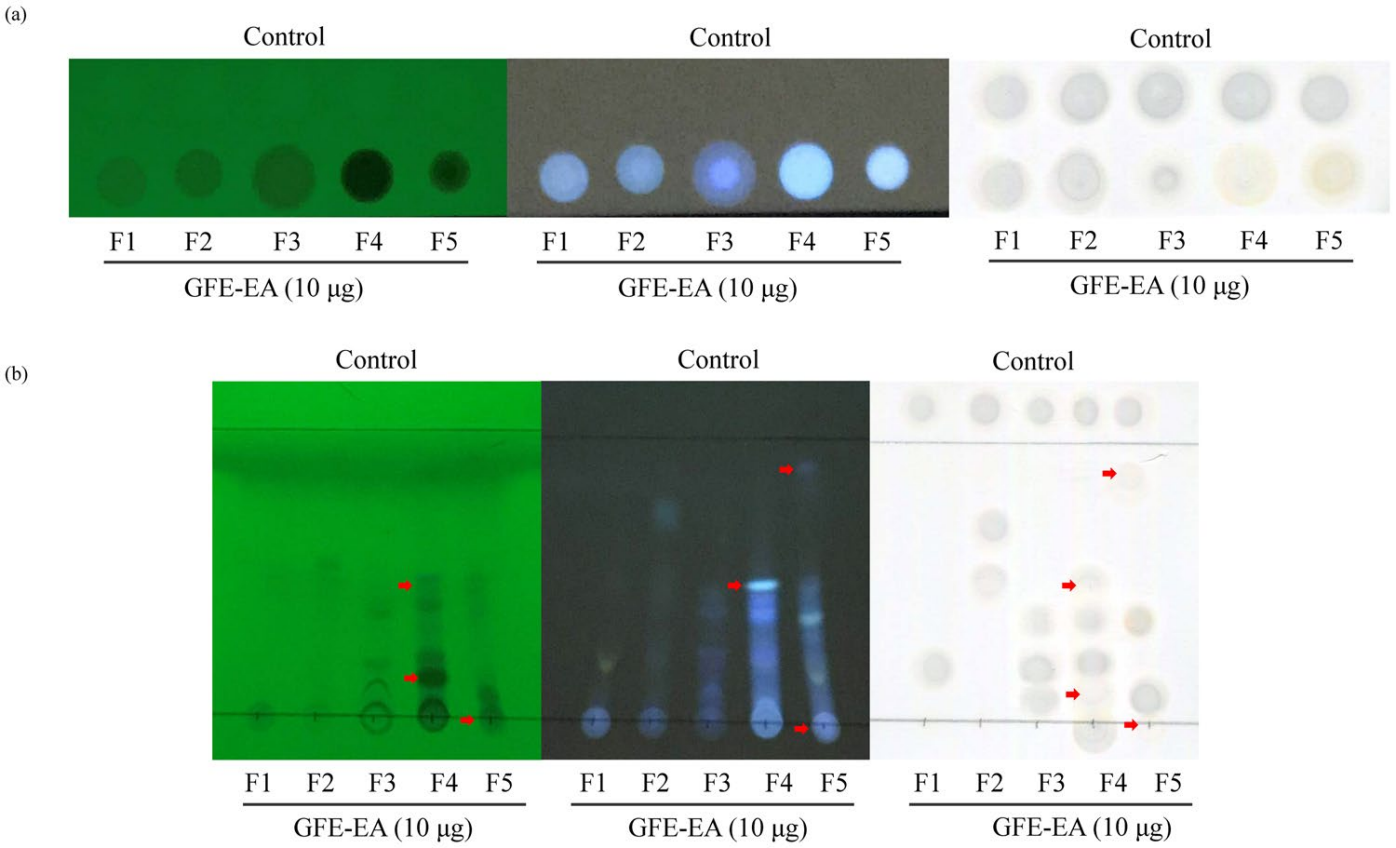
Tyrosinase-based TLC bioautography was employed to screen potential tyrosinase inhibitors from *G. formosanum* extracts. (**a**) results without TLC separation and (**b**) results with TLC separation.

In order to prove the concept for tyrosinase-based TLC allows researchers to conduct bioactivity evaluation and identify active compound(s) at once, anti-melanogenic activity of GFE-EA fractions purified via fractionating column was screened via tyrosinase-based TLC. In brief, five fractions of GFE-EA (F1–F5) was obtained after it purified by Sephadex LH-20, then 10 μg of each fraction was pipetted into the TLC plate, followed by addition of tyrosinase and L-DOPA. As presented in Fig. 5a, both F1 and F2 did not possess tyrosinase inhibitory activity. Besides, F3 to F5 did exert inhibitory effect on tyrosinase. It is obvious that depigmenting activity of F4 and F5 was higher than that of F3 by the naked-eye observation, showing the convenience of TLC bioautography for researchers. In addition, to separate and identify the major active constitute(s) in GFE-EA fractions, tested samples (F1–F5) were separated by TLC prior to tyrosinase inhibitor screening assay. Experimental results confirmed again that both F1 and F2 actually did not possess any potential tyrosinase inhibitor (Fig. 5b), and F3 to F5 authentically owned anti-melanogenic constitute(s); moreover, inhibition zone of certain spots on TLC illustrated more than one active compound in F4 and F5 (as red arrow indicated in Fig. 5b). These results imply that tyrosinase-based TLC enables researcher to identify potential compound(s) during bioactivity evaluation.

### Phenotype-based zebrafish verifies the depigmenting activity of candidate samples screened by tyrosinase-based TLC

**Figure 6:**
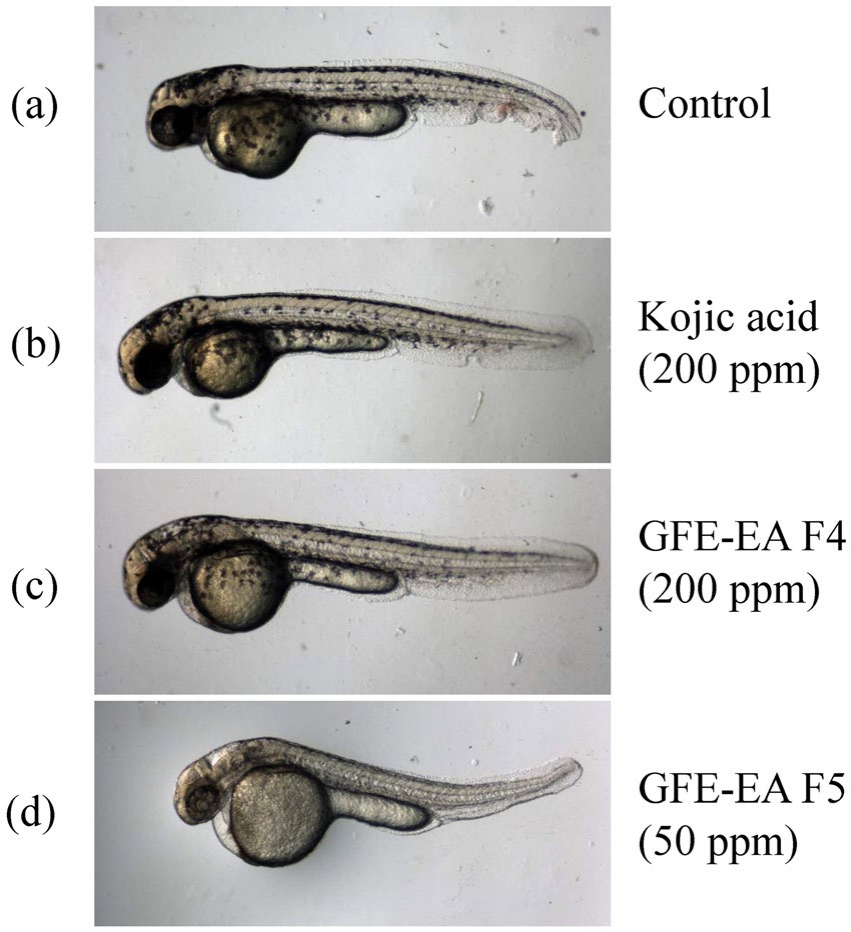
Depigmenting effect of GFE-EA fractions on zebrafish embryos. Representative picture of zebrafish embryos treated with various depigmenting ingredients after 55 hours post fertilization (48 hours treatment). (**a**) Zebrafish embryo without drug treatment as a control, (**b**) 200 ppm kojic acid as a positive control, (**c**) 200 ppm GFE-EA F4 (**d**) 50 ppm GFE-EA F5.

In zebrafish embryo testing, results demonstrated that both GFE-EA F4 (200 ppm) and F5 (50 ppm) exerted inhibition on zebrafish body pigmentation (Fig. 6), and the relative melanin amount of GFE-EA F4 and F5 treated groups were decreased 27.2 and 56.2% compared with control group, respectively. In addition, the relative melanin content of 200 ppm KA group, a positive control, was 38.46% of control group without treatment (Fig. 6). As a result, both GFE-EA F4 and F5 showed anti-melanogenic effect in tyrosinase-based TLC and zebrafish model, suggesting that tyrosinase-based TLC is a promising approach to depigmenting drugs screening.

## Discussion

Mushroom tyrosinase is widely used in screening tyrosinase inhibitors for skin lightening purpose owing to its commercially available^12^. Nowadays, TLC bioautography provides a bioactivity-guided purification and isolation^13^, where tyrosinase-based TLC autography is also developed to detect tyrosinase inhibitors; however, laborious preparation of materials and uneconomical use of enzyme in previous studies arose from an unsuitable substrate, L-tyrosine^14,15^. Although L-tyrosine and L-DOPA are generally applied to be a substrate for *in vitro* tyrosinase assay^16^, the inherent disadvantage of L-tyrosine for tyrosinase assay was poor water solubility^17^. Therefore, the addition of HCl was suggested to dissolve tyrosine^18^. Nevertheless, low pH caused by HCl will lead to enzyme inactivation. As shown in Supplementary Fig. S1, our preliminary test indicated that the substrate solution of tyrosinase assay containing 0.01 N HCl suppressed tyrosinase activity even through pH of solution has been adjusted to optimized level for tyrosinase activation (pH 6.8). Due to the low water solubility of tyrosine, all previous studies sprayed large amounts of tyrosine and tyrosinase on TLC plate in order to present clear inhibition zone of tyrosinase inhibitors^14,15^, leading to uneconomical use of valuable enzyme. In contrast, pipetting technique was employed in this study to improve those problems.

Sensitivity, stability and cost were the major consideration of an optimal experimental condition for tyrosinase-based TLC development. On that account, groups with 60 nmol L-DOPA were excluded for further experiment in light of assay stability and cost consideration (Fig. 2). Moreover, experimental results of 20 nmol L-DOPA groups demonstrated clearer inhibition zone on TLC than that of 5–10 nmol L-DOPA groups and possessed more economic property than 40 nmol L-DOPA groups (data not shown). In conclusion, experimental condition with 1 unit tyrosinase and 20 nmol L-DOPA was chosen for further experiment in view of the naked-eye observability, and it was worth noting that the amount of tyrosinase used was only 1 unit for further experiment, which is far less than previous study (4 to 8 units)^15^.

Rapid tyrosinase inhibitor screening was traditionally determined by colorimetric tyrosinase assay^19,20^. However, there is still room for improvement, especially for enzyme cost and tested compound consumption. In comparison, tyrosinase-based TLC broke through the detection limit of colorimetric assay for KA. Even though the amount of tested KA was only 0.0125 mg/mL, 21.83% tyrosinase inhibitory rate was sill determined via tyrosinase-based TLC platform (Fig. 3a). Besides, each reaction of tyrosinase-based TLC required far less tyrosinase (1 unit) compared to colorimetric method (9.6 units). Low enzyme consumption is its competitive edge compared with the existing tyrosinase assay platforms^19,20^.

In addition to low cost of enzyme, tyrosinase-based TLC can be employed to determine the anti-melanogenic activity of both hydrophobic and hydrophilic compounds. In traditional colorimetric assay, reaction must be implemented in buffer solution, impeding screening of tyrosinase inhibitor(s) from hydrophobic sample and misleading the experimental results. However, both hydrophobic and hydrophilic sample can be well attached to TLC plate surface for further bioactivity evaluation, avoiding a bias against hydrophobic compound(s).

Being able to repeat assay is regarded as a hallmark of drug screening technique^21^, enabling researchers to repeat the same experiment several times to verify their finding with low variation. Repeatability and reproducibility are the two components of measuring precision in an assay platform, and Gage R&R Nested study was widely employed to determine whether an analytical system is reliable^22^. Study varication of total Gage R&R in Tyrosinase-based TLC was 28.24%, verifying drug screening result of this platform can be well repeated and reproduced. In addition, if the whole experimental process can be further optimized, TLC bioautography could be expected to be more accurate and precise in quantification of tested sample’s activity. However, although we just used the simplest equipment and non-customized software to establish this technique, it was qualified to be a preliminary depigmenting drug screening system. In essence, this easy-to-follow technique enabled different operators to repeat and reproduce experiments with low variation (Tables 1 and 2).

To verify the applicability of tyrosinase-based TLC on anti-melanogenic drug screening, which was applied to screen various extracts of *G. formosanum* mycelium. As shown in Supplementary Fig. S2, experimental results indicated that the ethyl acetate fraction of *G. formosanum* mycelium ethanolic extract (GFE-EA) demonstrated the superior inhibitory activity toward tyrosinase compared with other fractions of *G. formosanum* mycelium ethanolic extract (hexane, butanol, and water fractions), which was consistent in our previous study^5^. Therefore, these results demonstrated that tyrosinase-based TLC was an authentic anti-melanogenic ingredient(s) screening system. Therefore, not only is tyrosinase-based TLC a superb technique to evaluate tyrosinase inhibitory activity of tested compound(s) with low-dose sample consumption, but also is used to identify certain active compound(s) in tested mixture. This technique provides insight into further purification and identification of active constitute(s).

Basically, this approach was developed in response to a need for more accurate information to assist us in identifying active compound(s) from GFE-EA. Traditionally, several aforementioned existing methods for anti-melanogenic drug screening just show whether tested extracts are active or not, but them can’t provide researcher information for further active compound(s) identification. In contrast, tyrosinase-based TLC enables us to separate various compound(s) of GFE-EA fractions by TLC chromatographic separation, and those compounds can be visualized by long and short-wave UV (365 and 254 nm, respectively) (Fig. 5b). Consequently, we not only demonstrated that GFE-EA F4 and F5 exerted depigmenting activity, but also showed which spots were active (Fig. 5b). This information enables us to identify active compound(s) in a mixture more accurate, avoiding unnecessary labor and sample consumption. Moreover, the principle of TLC is similar to high performance liquid chromatography (HPLC), so data from tyrosinase-based TLC can assist us in purifying compound(s) through HPLC. In addition, electrospray-assisted laser desorption/ ionization mass spectrometry (ELDI-MS) had been developed to characterize compound(s) separated in TLC directly^23^, suggesting that the combination of tyrosinase-based TLC and ELDI-MS might be the most powerful technique to screening active compounds in mixtures in the future.

The last step that must be examined is the correlation between tyrosinase-based TLC and animal model for testing anti-melanogenic drug. In view of this, phenotype-based zebrafish platform, a promising animal model for anti-melanogenic drug screening^24^, was performed to verify the anti-melanogenic effect of GFE-EA F4 and F5 screened by tyrosinase-based TLC. The anti-melanogenic activity of GFE-EA F4 and F5 were consistency between tyrosinase-based TLC and zebrafish platforms (Fig. 6), which showed that tyrosinase-based TLC is a promising technique for anti-melanogenic drug screening.

It is worth noting that animal test for cosmetics ingredients has been banned by European union (EU) since 2009, and the sale of animal tested cosmetics has also been banned from March 2013^25,26^. Furthermore, more and more countries have legislated against animal testing for cosmetic products; for instance, Taiwan has become the first Asian country to ban cosmetics animal testing in 2016. Thus, the non-animal alternatives for anti-melanogenic drug screening are urgently required. Since the correlation of depigmenting activity of tested sample between tyrosinase-based TLC and zebrafish model was consistent, this TLC bioautographic assay totally meets the need of ethnic cosmetics development.

Currently, this platform was applied to screen action fraction(s) from 20 GFE-EA fractions purified via silica gel column, and one active fraction has been indicated (Fig. S3). Furthermore, this active fraction was fractionated into 30 sub-fractions through Sephadex^®^ LH-20 chromatography, and one sub-fraction exhibited highest depigmenting activity has also been identified by tyrosinase-based TLC (Fig. S3 and S5). It was worth noting that the whole depigmenting activity assay for 50 candidate samples was completed within few hours (one assay just takes 25 minutes to complete), suggesting the high-throughput potential of tyrosinase-based TLC.

## Conclusion

Tyrosinase-based TLC autography is a simple, economic, and high-throughput screening technique for the rapid determination of tyrosinase inhibitory activity of tested compound(s). It is noticeable that the assay process of this platform can be implemented within 30 minutes, enabling researchers to screen potential melanogenesis inhibitor efficiently. Gauge R&R Nested study also indicated that tyrosinase-based TLC is a well-repeatable and high-reproducible assay system for different researchers to evaluate melanogenesis inhibitory activity of tested drug with low variation. Better yet, the low-cost feature of this technique enables users to conduct large-scale drug screening at keen prices (100 assays/US$0.4). In contrast, commercial tyrosinase inhibitor screening kit (BioVision Inc., Milpitas, CA, USA) demands an exorbitant price (100 assays/US$695.00, latest accessed April 20, 2017)^27^. And the most striking feature of tyrosinase-based TLC is that it can enable researchers to identify the major bioactive ingredient(s) in a tested mixture, accelerating discovery of novel anti-melanogenic drug(s).

Most important, this technique is a compound-oriented as well as bioactivity-guided assay. For instance, in this present study, GFE-EA F4 and F5 was identified as melanogenesis inhibitor from several fractions of GFE-EA via tyrosinase-based TLC analysis. As shown in Fig. 5a, it is clear that the inhibition zone of GFE-EA F4 and F5 on melanin spot were lighter than other fractions, and the numbers of tyrosinase inhibitors in them could be estimated through TLC separation before anti-melanogenic activity determination. In addition to that, results of phenotype zebrafish embryo model also confirmed that GFE-EA F4 and F5 possessed melanogenic inhibitory property to verify the reliability of tyrosinase-based TLC. Moreover, high correlation of screening results between TLC bioautography and animal model, demonstrating that the possibility to replace cosmetics animal testing by our technique to accord with the EU cosmetics regulation, for novel depigmenting ingredients evaluation.

Nowadays, characterization of organic compound(s) in TLC plate by ELDI-MS have been established^23^, suggesting that combination of tyrosinase-based TLC and ELDI-MS in the future would be a more efficient way to screen and identify novel anti-melanogenic drug. In conclusion, tyrosinase-based TLC autography is the most rapid and economic platform for anti-melanogenic drug screening, providing a new insight and non-animal alternatives into novel melanogenesis inhibitor discovery.

## Material and Methods

### Preparation of L-tyrosine, L-DOPA and tyrosinase stock solution

Lyophilized mushroom tyrosinase was purchased from Sigma Inc. (St Louis, MO, USA), and various concentration of tyrosinase stock solution was concocted by dissolving tyrosinase powder in 20 mM phosphate buffer (pH 6.8). L-tyrosine and L-DOPA were obtained from Sigma Inc. and dissolved in phosphate buffer (PB) as well.

### Comparison of pigmentation effect of L-tyrosine and L-DOPA on TLC

Aluminum TLC (TLC silica gel 60 F_254_, Merck, Darmstadt, Germany) was employed to be a platform for tyrosinase assay. Pigmentation effect of L-tyrosine and L-DOPA were compared on TLC. In brief, different groups with various amount (20–60 nmol) of L-tyrosine and L-DOPA dissolved in PB were pipetted onto TLC plate and subsequently dried at room temperature for 5 minutes. After that, each spot on TLC with various amount of L-tyrosine and L-DOPA was incubated with tyorinase (2 units) at room temperature for 10 minutes. Finally, color depth of each spot was scanned by scanner (Perfection V37, EPSON, Nagano, Japan) and quantified by software to compare pigmentation effect of L-tyrosine and L-DOPA on TLC.

### Tyrosinase-based TLC assay procedure

In short, this assay was composed of three steps as follows: First, 2 μl KA solution (TCI Chemical, Tokyo, Japan) was pipetted into TLC plate, followed by air drying for 5 minutes. Second, KA was incubated with 1 μl PB containing tyrosinase for 5 minutes. Third, 2 μl L-DOPA was dropped onto the same area. After 15-minutes incubation, inhibitory zone of each spot was scanned by scanner (Perfection V37, EPSON) and analyzed by software for further quantification.

After optimization of substrate and enzyme amount, 20 nmol L-DOPA and 1 unit tyrosinase were employed to conduct tyrosinase-based TLC assay for evaluation of tyrosinase inhibitory activity of KA and *G. formosanum* extracts.

### Image quantification of tyrosinase-based TLC assay

To quantify signals of inhibition spots on TLC, image processing was conducted by Image J version 1.8.0, an open source Java program created by Wayne Rasband of National Institutes of Health (Bethesda, MD, USA). After quantification, color depth of each spot on TLC was presented as a peak.

To evaluate pigmentation of groups with various amount of substrate or enzyme, pigmentation level was calculated as (%) = (A/B) × 100, where A and B represent the area of peak of group under different conditions, respectively. For anti-tyrosinase activity evaluation, tyrosinase inhibitory activity of tested compound was calculated as (%) = (C/D) × 100, where C and D represent the area of peak of group with and without tested compound, respectively.

### Tyrosinase colorimetric assay

To evaluate tyrosinase inhibitory rate of different concentration of KA, 20 μl tyrosinase (480 units/mL) was mixed with 160 μl KA dissolved in PB buffer for 10 minutes, and subsequently mixed with 20 μl L-DOPA (0.92 mM) for 20 minutes at room temperature. Tyrosinase activity was determine at 475 nm using Thermo Multiskan GO microplate spectrophotometer (Thermo Scientific, Waltham, MA, USA), and tyrosinase inhibitory rate was calculated as (%) = [1 − (A– B)/(C − D)] × 100, where A and B represent the absorbance of KA group with and without tyrosinase respectively; C and D represent the absorbance of control group with and without tyrosinase respectively^5^.

### Repeatability and reproducibility

To determine the adequacy of measurement system, Gage R&R (repeatability and reproducibility) Nested study based on analysis of variance (ANOVA) was conducted using Minitab version 16 software (Minitab Inc., State College, PA, USA).

Gauge variability was calculated by following equation:

1 $${\sigma }_{Measurement\,error}^{2}={\sigma }_{gauge}^{2}={\sigma }_{repeatability}^{2}+{\sigma }_{reproducibility\,}^{2}$$

where

2 $${\sigma }_{repeatability}^{2}=M{S}_{error}$$

and

3 $${\sigma }_{reproducibility}^{2}=\frac{M{S}_{operators}-M{S}_{parts}}{{n}_{parts}\times {n}_{repetitions}}$$

### Preparation of *Ganoderma formosanum* mycelial extracts

*Ganoderma formosanum* were cultured at 25 °C in biomass-oriented medium comprising 65 g glucose, 7.5 g yeast extract, 0.88 g KH_2_PO_4_, 0.5 g MgSO_4_·7H_2_O, and 0.05 g vitamin B1 per liter of deionized water, at initial pH 6.5 with rotation at 120 rpm^28^. After 9-days fermentation, *G*. *formosanum* mycelium were harvested and lyophilized. The lyophilized mycelia were extracted with 95% ethanol, and then subsequently fractionalized with water, hexane, ethyl acetate, and butanol to obtain various fractions of *G*. *formosanum* mycelial ethanol extract. Furthermore, the certain fraction exerting tyrosinase inhibitory effect was further purified via Sephadex LH-20 column chromatography (GE Healthcare, Uppsala, Sweden) with 100% methanol elution to obtain sub-fractions for further study.

### Tyrosinase-based TLC assay for *G. formosanum* mycelial extracts

Ten μg of *G. formosanum* mycelial extracts (GFME) were dissolved in methanol and pipetted into a TLC plate (Merck), and standing 5 minutes for evaporation of organic solvent. Furthermore, 1 unit of mushroom tyrosinase was pipetted into the spot of GFME. After 5-minutes incubation, detection was carried out with 20 nmol L-DOPA for 15-minutes reaction to evaluate anti-melanogenic effect of GFME. What’s more, for the identification of the certain compound(s) of GFME with tyrosinase inhibitory activity, TLC chromatographic separation must be conducted prior to tyrosinase-based TLC assay. After a separation was complete, spots of GFME was visualized through long and short-wave UV (365 and 254 nm, respectively) using UVGL-25 UV lamp (UVP, CA, U.S.A), and tyrosinase inhibitory activity of each spot was determined by aforementioned tyrosinase-based TLC assay.

### Zebrafish embryos maintenance

Zebrafish embryos were purchased from TechComm Zebrafish Core, National Taiwan University and cultured in Danieau’s Medium [0.45 mM HEPES (pH7.6), 0.036 mM MgSO_4_, 0.054 mM Ca(NO_3_)_2_, 5.22 mM NaCl, 0.063 mM KCl.] at 28 °C^5^. The maintenance of zebrafish embryos followed the guidelines for the use of laboratory animals and was approved by the Institutional Animal Care and Use Committee, National Taiwan University (Approval number: NTU105-EL-00082).

### Phenotype-based evaluation of zebrafish embryos

Synchronized embryos (7 hours post fertilization, hpf) were collected and assigned to 96-well plates, and each well comprises 2 embryos and 80 μl Danieau’s Medium contained tested extracts. After treatment with tested extracts for 48 hours, zebrafish embryo was mounted in 3% methyl cellulose on the depression slide and taken photographs via stereomicroscope (Olympus-SZ61, Olympus optical Co, Tokyo, Japan) to evaluate the anti-melanogenic effects of tested extracts. The animal use protocol aforementioned has been reviewed and approved by the Institutional Animal Care and Use Committee, National Taiwan University (Approval number: NTU105-EL-00082).

### Determination of relative melanin contents of zebrafish embryos

To determine relative melanin content of zebrafish embryos, 55 hpf zebrafish embryos was lysed with 1 N NaOH at 100 °C for 1 hour, followed by centrifugation. Melanin level of each zebrafish group was determined by reading the absorbance at 405 nm through spectrophotometer (Thermo Multiskan GO, Thermo Fisher Scientific CO., Ltd, Waltham, MA, USA), then referred to melanin standard curve. For normalization of melanin content, total protein of each zebrafish group was determined via Bradford assay (Bio-Rad, Richmond, CA, USA) to calculate relative melanin content between various groups^5^.

### Statistical analysis

All of the data in this study were performed in duplicates or triplicates at least to ensure the credibility of our experimental results. Significant of experimental results was obtained by Student’s t-test, and all data were presented as the means ± SD.

## Electronic supplementary material

Supplementary information
