# Supplementary material for: Tyrosinase-based TLC Autography for anti-melanogenic drug screening
Source: Sci Rep. 2018 Jan 10;8:401. doi: 10.1038/s41598-017-18720-0 (PMC5762723; doi:10.1038/s41598-017-18720-0)
Supplement: Supplementary file 1 — Supplementary information [file 41598_2017_18720_MOESM1_ESM.docx]

**Tyrosinase-based TLC Autography for Anti-Melanogenic Drug Screening.**

Kai-Di Hsu^1§^, Yu-Hin Chan^1§^, Hong-Jhang Chen^2^, Shi-Ping Lin^1^, Kuan-Chen Cheng^1, 2, 3^

1. Institute of Biotechnology, National Taiwan University, Taipei 10617, Taiwan
2. Graduate Institute of Food Science Technology, National Taiwan University, Taipei 10617, Taiwan
3. Department of Medical Research, China Medical University Hospital, China Medical University, Taichung, Taiwan.

§ Kai-Di Hsu and Yu-Hin Chan contributed equally to this study.

* Corresponding author. 1, Sec 4, Roosevelt Rd., Taipei 10617 Taiwan;

Tel.: +1 886233661502; fax: +1 886223620847.

E-mail addresses: [kccheng@ntu.edu.tw](mailto:kccheng@ntu.edu.tw) (K.C. Cheng)

**Supplementary information**

**Results**

As shown in Fig. S1, the addition of hydrochloric acid (HCl) to phosphate buffer for tyrosinase assay lowered tyrosinase activity. Moreover, the addition of HCl was suggested to dissolve tyrosine. Therefore, tyrosine is not a suitable substrate for development of tyrosinase-based TLC.

In our previous study, ethyl acetate fraction of *G. formosanum* mycelium ethanolic extract (GFE-EA) was found to exert higher tyrosinase inhibitory activity compared to other fractions of *G. formosanum* mycelium ethanolic extract. Consistent result was reproduced in tyrosinase-based TLC bioautography, demonstrating this technique was authentic to screen depigmenting ingredient(s) (Fig. S2).

In order to evaluate the high-throughput property of this technique, tyrosinase-based TLC was employed to assist us in identifying active ingredient(s) from GFE-EA. In brief, 20 GFE-EA fractions were obtained after silica gel chromatography, and one fraction dubbed GFE-EA 50% exhibited the greatest tyrosinase inhibitory activity (Fig. S3). To further purify the tyrosinase inhibitor(s), GFE-EA 50% was fractionated by Sephadex^®^ LH-20 chromatography, and F4-2-5 of GFE-EA 50% (50% F4-2-5) demonstrated the strongest anti-melanogenic effect among 30 sub-fractions (Fig. S4). To confirm the tyrosinase inhibitory activity of 50% F4-2-5, inhibition spots on TLC was quantified by ImageJ. As shown in Fig. S5, ten micrograms of 50% F4-2-5 showed 50.57 **±** 3.75% of tyrosinase inhibitory rate. Taken together, tyrosinase-based TLC autography allowed operator to identify 50% F-4-2-5 from 50 candidate fractions within a few hours, suggesting its high-throughput potential.

Although GFE-EA has been proved its skin lightening effect in our previous study, the compound(s) responsible for anti-melanogenic activity remains unknown. As 50% F4-2-5 exerted anti-melanogenic activity, and single spot was shown on TLC plate after TLC chromatographic separation (data not shown), 50% F4-2-5 was considered as pure tyrosinase inhibitor candidate responsible for the anti-melanogenic activity of GFE-EA. Furthermore, results of mass spectrum demonstrated molecular weight distribution of 50% F4-2-5 (Fig. S6), which is informative for further identification of active compound(s) of GFE-EA.

As an anti-melanogenic drugs screening platform, tyrosinase-based TLC shows convenience and advantages in identification of *G. formosanum* active compounds. Besides, only ten μg of each fraction was required for each tyrosinase-based TLC assay. It was worth noting that only few mg of 50% F-4-2-5 purified from total 5 kg of *G. formosanum* mycelium, hence compound-saving property during bioactivity determination in tyrosinase-based TLC is important for following mass spectrometry and nuclear magnetic resonance analysis.

**Figure Caption**

**Figure S1. The influence of hydrochloric acid (0.01N) on tyrosinase activity with and without pH adjustment for tyrosinase.** Tyrosine was replaced with L-DOPA as the substrate in this 10-minutes assay in light of naked-eye observability.

**Figure S2. Inhibitory effects of *G. formosanum* ethanolic extracts (GFE) on tyrosinase activity.** E= ethanolic extract of G. formosanum, H=Hexane fraction of GFE, EA=ethyl acetate fraction of GFE, B=Butanol fraction of GFE, W=Water fraction of GFE.

**Figure S3. Identification of GFE-EA fractions with anti-melanogenic activity.**

**Figure S4. Identification of sub-fractions of GFE-EA 50% fraction with anti-melanogenic activity.**

**Figure S5. Comparison of F4-2-5 sub-fraction and kojic acid for inhibiting tyrosinse activity.**

**Figure S6. Mass spectrometry analysis of F4-2-5 sub-fraction.**

Figure S1.


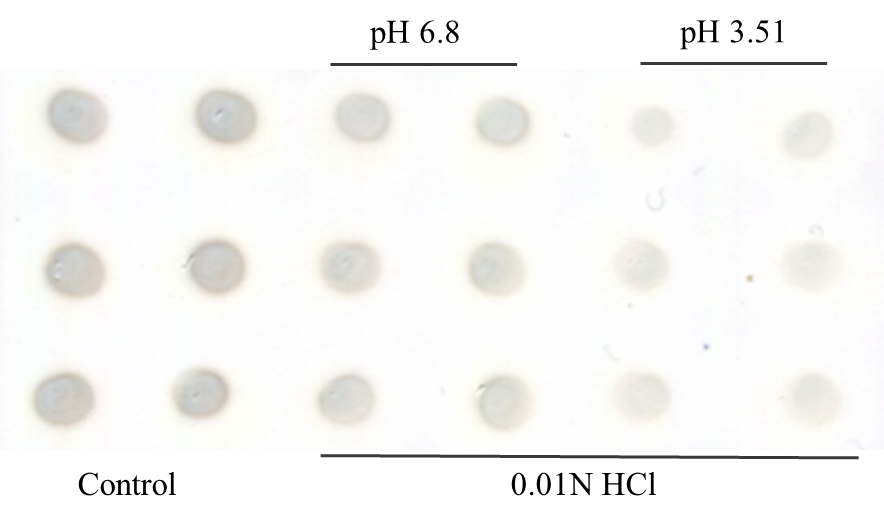


Figure S2.


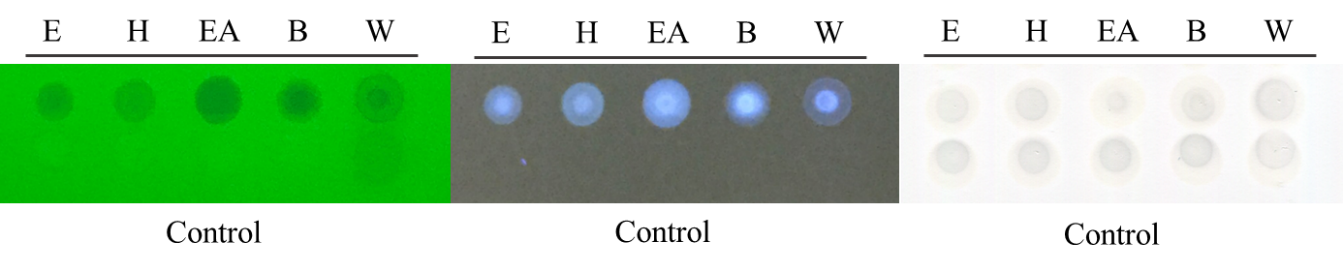


Figure S3.


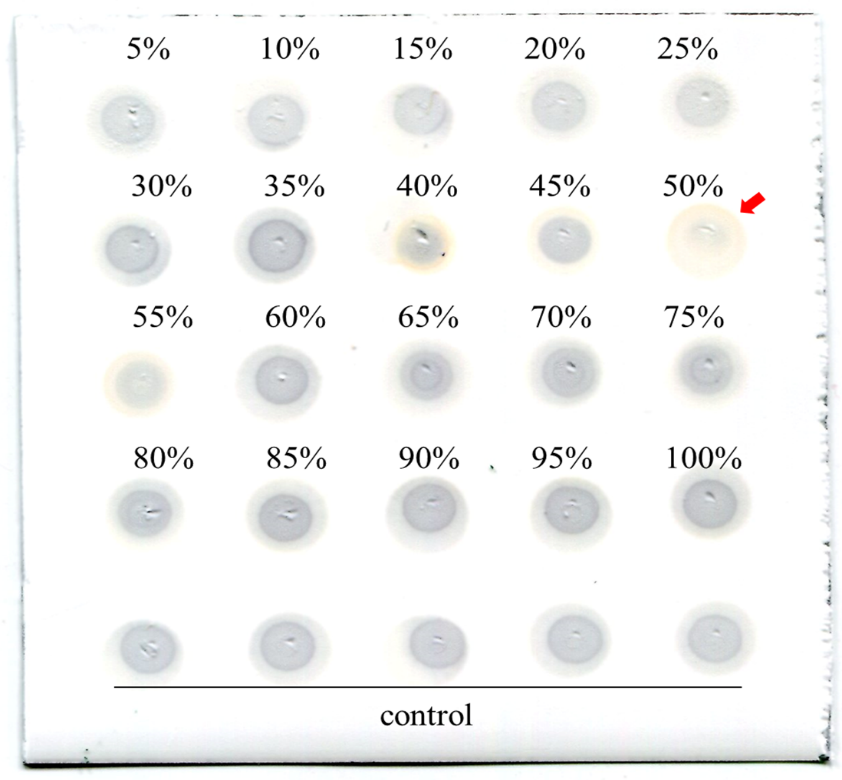


Figure S4.


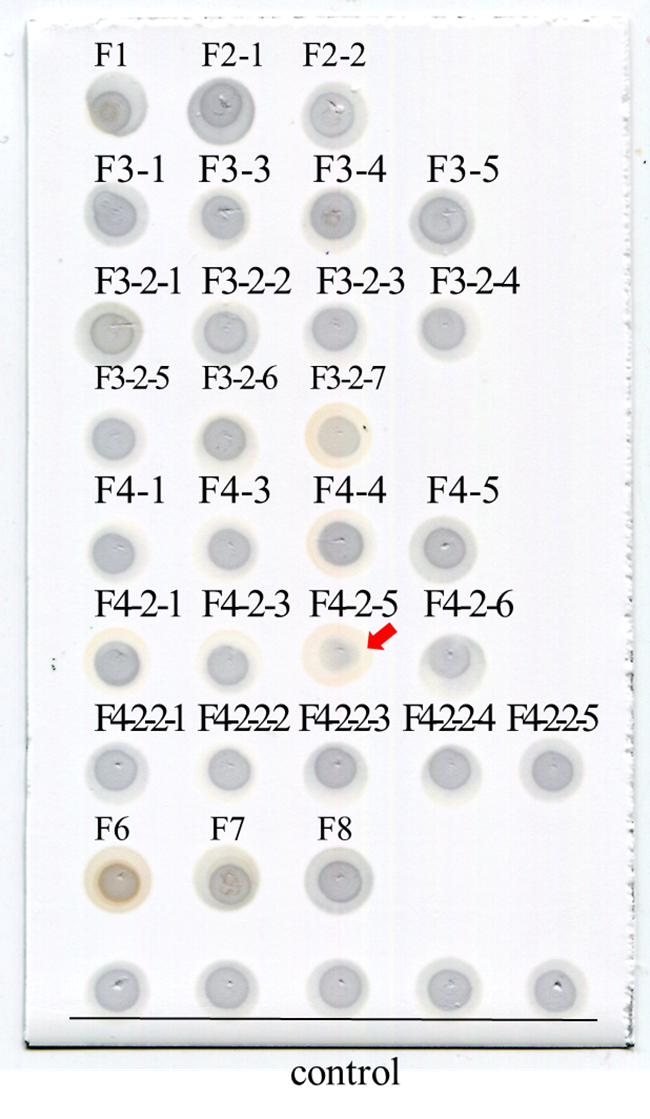


Figure S5.


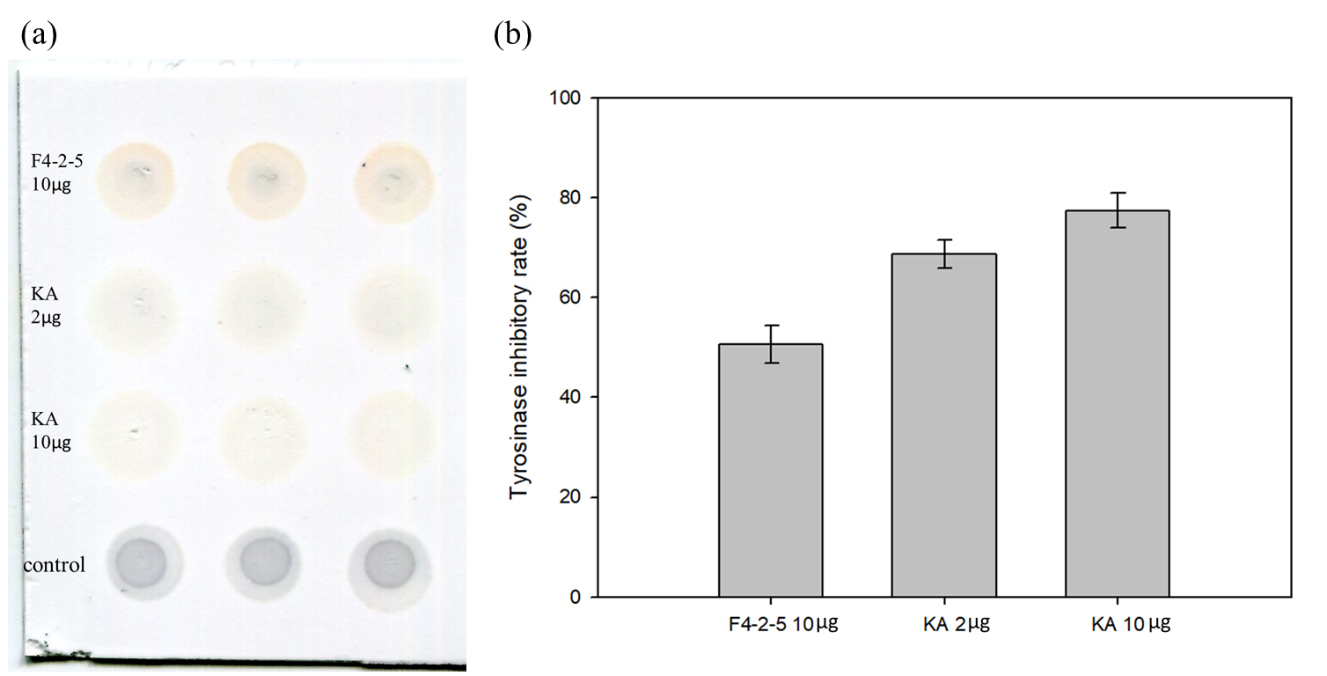


Figure S6.


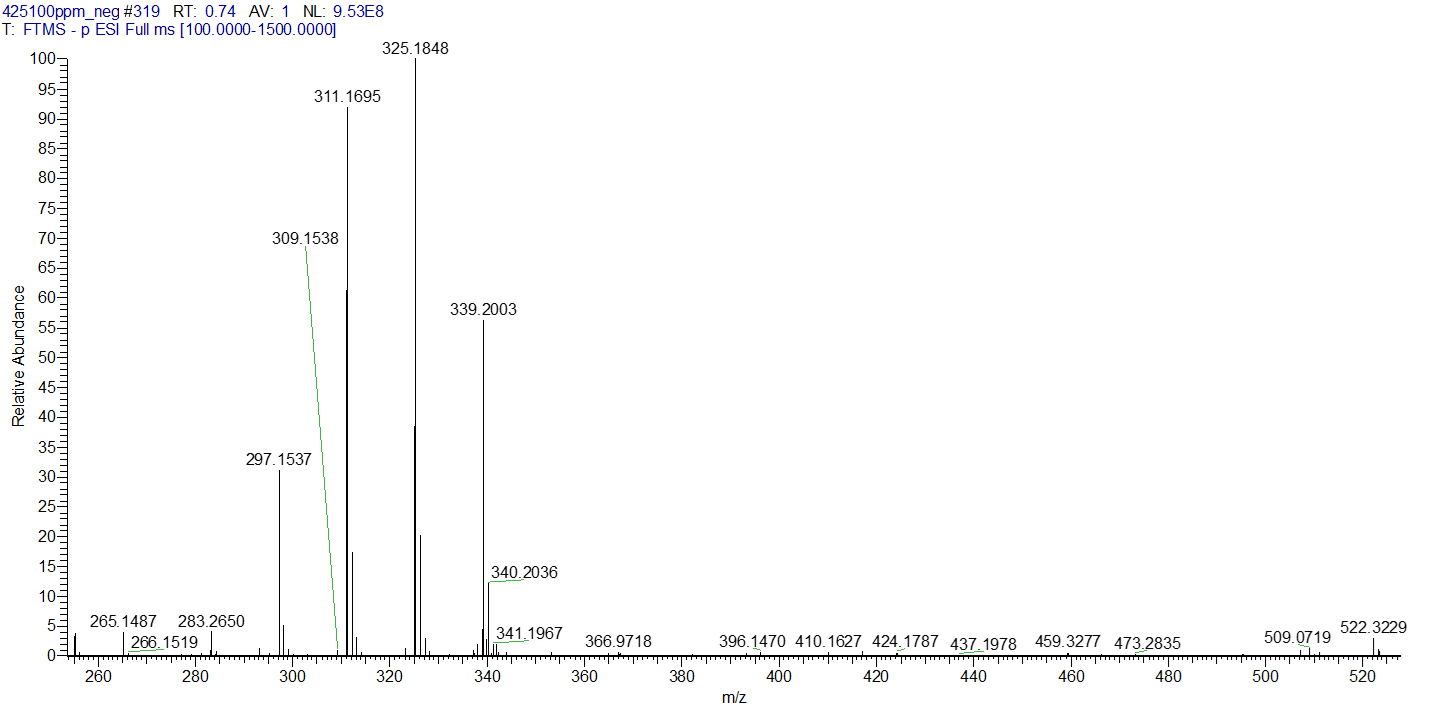


**Materials and Methods**

Eighteen mg of GFE-EA was purified by silica gel column chromatography with gradient solvent composed of *n*-hexane and ethyl acetate (10:0, 9:1, 8:2, 7:3, 6:4, 1:1, 4:6, 3:7, 2:8, 1:9, 0:10, v/v) to obtain GFE-EA fractions. Furthermore, GFE-EA fraction 50% (GFE-EA 50%) was fractionated by Sephadex^®^ LH-20 column to obtain sub-fractions, followed by tyrosinase-based TLC assay. In brief, ten μg of GFE-EA fractions and sub-fractions were dissolved in methanol and pipetted onto a TLC plate (Merck), standing it for 5 minutes to evaporate organic solvent. Then, 1 unit of mushroom tyrosinase was pipetted onto the spot of *G. formosanum* mycelial extracts. After 5-minutes incubation, detection was carried out with 20 nmol L-DOPA for 15 minutes to identify the active compound.
